# Frontiers in Restorative Dentistry Biomaterials and Endodontic Instruments

**DOI:** 10.3390/ma16020475

**Published:** 2023-01-04

**Authors:** Claudio Poggio, Simone Gallo

**Affiliations:** 1 Unit of Restorative Dentistry, Department of Clinical, Surgical, Diagnostic and Pediatric Sciences, Section of Dentistry, University of Pavia, 27100 Pavia, Italy; 2 Unit of Orthodontics and Pediatric Dentistry, Department of Clinical, Surgical, Diagnostic and Pediatric Sciences, Section of Dentistry, University of Pavia, 27100 Pavia, Italy

Restorative dentistry deals with the prevention, diagnosis, and treatment of pathological conditions affecting the teeth, to restore their function and aesthetics.

Teeth are complex structures made of unique hard tissues in the body, i.e., the enamel, dentin, and cementum, surrounded by soft tissues and the alveolar bone. The enamel constitutes the outer part of the dental crown, and owing to its hardness, as the hardest tissue in the body, it represents the first barrier from external harmful agents such as bacterial pathogens and acidic substances, which might compromise tooth integrity [1].

In addition to the preservation of the external surface of the tooth, every effort should be made to maintain the condition of the inner part of the tooth, i.e., dental pulp, where a unique connective tissue guarantees vascularization and innervation. Deep decay and dental trauma are generally the most frequent conditions affecting dental pulp, as a result of which tooth vitality can be compromised. In these cases, in fact, crown restoration can be performed only after removing pulp tissues from the tooth and filling the pulpal space with specific endodontic materials [2].

Since the integrity of the tooth should be the primary goal for both the patient and the dentist, the major focus should be on preventive strategies against caries development [3,4,5]. In particular, the use of sealants to fill the pits and fissures of first permanent molars is regarded as one of the most relevant preventive measures in addition to topical prophylaxis with fluoride.

Nevertheless, dental caries, periodontal diseases, traumatic injuries, and conditions resulting from genetics, such as amelogenesis imperfecta or molar–incisor hypomineralisation (MIH), represent important factors that could cause partial damage to the dental structure or eventually tooth loss at any age. Thus, the need arises for clinicians and researchers to develop new strategies based on innovative biomaterials able to mimic lost dental tissues with the highest respect to the residual tooth structure [6,7,8,9,10].

Against the backdrop of these considerations, this Special Issue entitled “Frontiers in Restorative Dentistry Biomaterials and Endodontic Instruments” aims to collect studies, including in vitro investigations, clinical trials, case series, case reports, and reviews, that contribute new knowledge to the field of dental biomaterials in restorative dentistry and endodontics. The topics considered in this Special Issue include, but are not limited to, the following themes: new adhesive strategies, bioactive materials, sealants, composite resins, ceramics, enamel and dentin pretreatments, endodontic instruments, endodontic materials, regenerative dentistry, and dental tissue engineering.

## Data Availability

Not applicable.
